# Biodegradable magnesium screw, titanium screw and direct embedding fixation in pedicled vascularized iliac bone graft transfer for osteonecrosis of the femoral head: a randomized controlled study

**DOI:** 10.1186/s13018-023-04012-z

**Published:** 2023-07-22

**Authors:** Jiahao Sun, Zhipeng Li, Shaowei Liu, Tianwei Xia, Jirong Shen

**Affiliations:** 1 Department of Orthopaedics and Traumatology, Affiliated Hospital of Nanjing University of Chinese Medicine, Nanjing, 210029 Jiangsu China; 2 Department of Radiology, Affiliated Hospital of Nanjing University of Chinese Medicine, Nanjing, 210029 Jiangsu China

**Keywords:** Osteonecrosis of the femoral head (ONFH), Pedicled vascularized iliac bone graft transfer (PVIBGT), Biodegradable magnesium internal fixation screw, Dynamic contrast-enhanced magnetic resonance imaging (DCE-MRI), Hip preservation

## Abstract

**Background:**

The use of degradable magnesium screws to fix the bone flap implanted in the treatment of femoral head necrosis has achieved preliminary good therapeutic results. However, there is no conclusive evidence in the study to demonstrate whether biodegradable magnesium screws promote angiogenesis and no comparison has been made between degradable magnesium screws and traditional screws.

**Objective:**

To investigate the clinical efficacy and safety of biodegradable magnesium screws in pedicled vascularized iliac bone graft transfer (PVIBGT) for osteonecrosis of the femoral head (ONFH).

**Materials and methods:**

A total of thirty-six patients (37 hips) with ONFH were recruited from March 2020 to July 2022. The study used a single-blind method, and patients who underwent PVIBGT were randomized into three groups: 12 patients (12 hips) were fixed with biodegradable magnesium screws (Group A), 12 patients (13 hips) were fixed with titanium screws (Group B), 12 patients (12 hips) were directly embedded (Group C). The operating time and the length of stay were recorded. Harris scores, radiological examinations (X-ray, CT, DCE-MRI), blood and serum tests were conducted before and after surgery. The gas yield and degradation rates of the magnesium screws were measured at the 3-months and 6-months post-operative follow-ups in Group A.

**Results:**

There was no statistically significant difference among these three groups in terms of types, gender, age, course of disease, surgical side, operation time, the length of stay (*P* > 0.05). All patients were followed up for 6 months. The mean Harris scores were higher in all groups 6 months after surgery (*P* < 0.05). The rates of excellent and good outcomes were 66.7%, 46.2%, and 33.3% in Groups A, B, and C, respectively. PVIBGT and magnesium screws can improve the blood supply of the femoral head via DCE-MRI evaluation. Two patients with poor incision healing received prompt treatment and subsequently recovered well. No adverse events, such as hip infection or deep vein thrombosis, were reported in the patients. The patients had good biocompatibility of magnesium screws, and no fracture of the magnesium screws was observed in Group A. Liver and kidney functions (including serum magnesium) were within normal ranges. The area of the intermuscular air space was 0 cm^2^ in follow-ups. The degradation rate of the biodegradable magnesium screws was approximately 10.32% at the 3-months follow-up and 13.72% at the 6-months follow-up.

**Conclusions:**

PVIBGT has a positive effect, especially with regard to improving blood supply of the femoral head. The fixation of biodegradable magnesium screws is reliable and safe in PVIBGT, and promote angiogenesis.

## Introduction

Osteonecrosis of the femoral head (ONFH) is a joint disease with high morbidity and the disability rate that affects young adults [1–3]. Considering the limitation of artificial joint, hip preservation is necessary for proper patients.

The pedicled vascularized iliac bone graft transfer with the ascending branch of the lateral femoral artery (PVIBGT) is an important method in hip preserving [4–6]. It provides stable mechanical support in necrotic area, improves local blood supply, delays total hip arthoplasty and prevents deterioration of patients’ conditions [7, 8]. The stability of the iliac flap graft with the ascending branch of the lateral spinous femoral artery is critical to the success of the surgery [9]. The traditional method of fixing the implanted flap is anatomical fitting (such as direct embedding bone graft). However, this method can lead to displacement of the iliac bone graft, and this displacement can damage the surrounding vessels. Common screws (e.g. titanium screw) are not biodegradable, and will lead to inflammation. Moreover, titanium screws cannot promote osteogenesis and angiogenesis by themselves. Thus, biodegradable screws which are capable of boosting osteogenesis and angiogenesis are very important. Zhao Dewei's team pioneered the use of biodegradable magnesium screws for the fixation of bone grafts in the treatment of ONFH, and initially achieved good results [10, 11]. However, the study by Zhao's team did not effectively prove whether the biodegradable magnesium screw had an angiogenic effect. They also did not compare the biodegradable magnesium screw with common screws; their comparison only focused on directly embedded bone graft. Thus, further clinical trials are urgently needed to investigate the use of biodegradable magnesium screws.

We set up a trail group (Group A) and two control groups (Groups B and C) to investigate the efficacy and safety of biodegradable magnesium screws in the treatment of ONFH with PVIBGT. Moreover, DCE-MRI, a new and effective method, was used to evaluate the blood supply of the femoral head in PVIBGT.

## Materials and methods

### Flowchart of study protocol

See Fig. 1.

**Fig. 1:**
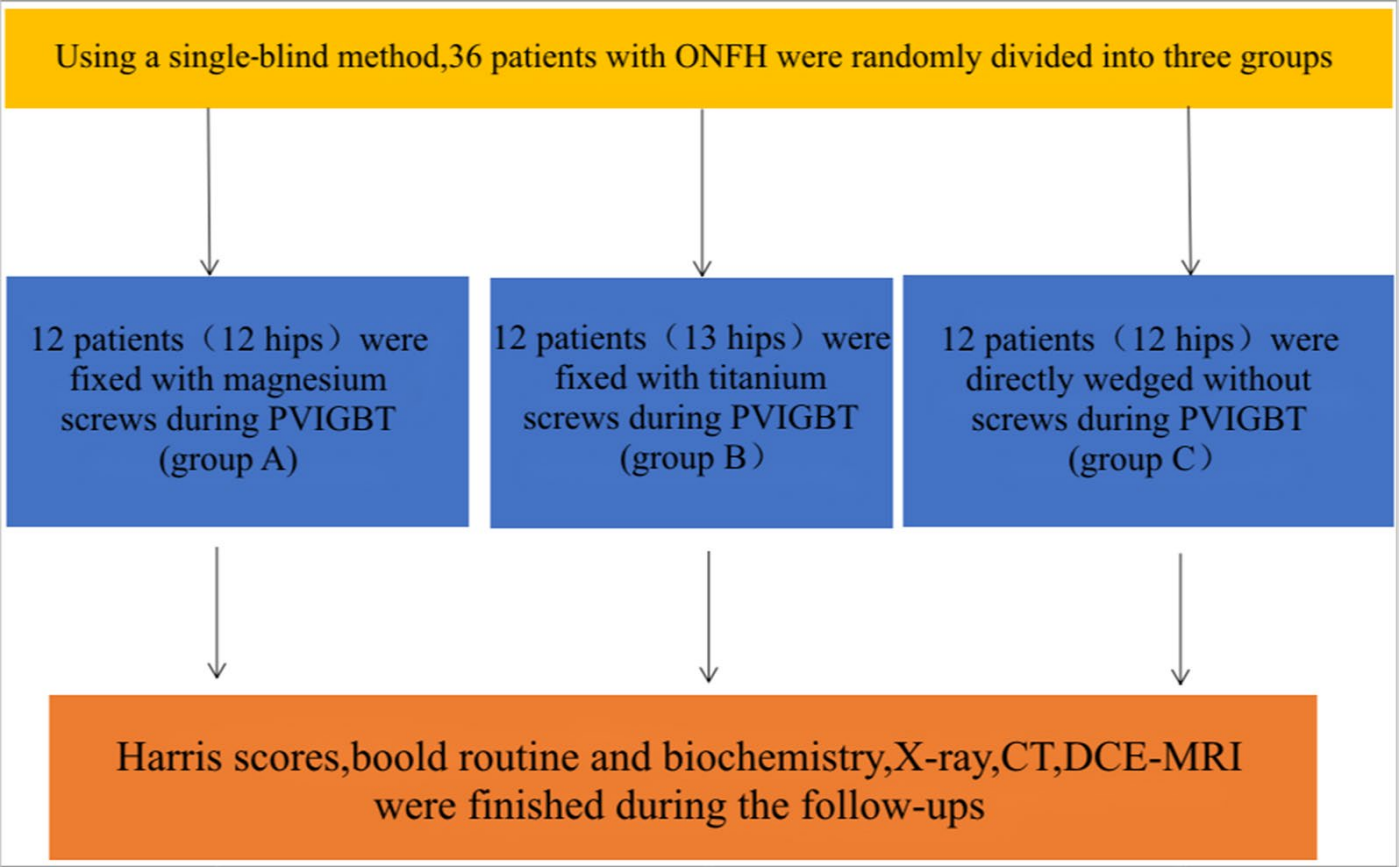
Flowchart of the study

### Patient selection criteria

#### Inclusion criteria

Patients must meet all of the following conditions.

1. Age range: 18–55 years old (including 18 and 55 years old).
2. Patients were in ARCO (Association Research Circulation Osseous) II and III.
3. Patients understood the objective of the clinical trial, signed the informed consent form voluntarily, and agreed to complete the entire research process according to the protocol.

#### Exclusion criteria

Patients were excluded if they met one of the following conditions.

1. Accompanied by an acute or serious infection;
2. Patients had hypermagnesemia;
3. Patients used glucocorticoids in the past three months;
4. Patients with serious cardiovascular and cerebrovascular diseases, diabetes, coagulation dysfunction, mental and nervous system diseases, serious osteoporosis, calcium and phosphorus metabolism disorders;
5. Patients abused drugs;
6. Patients were experiencing an acute attack of chronic diseases;
7. Patients with malignant diseases and pre-survival periods were less than 12 months;
8. Patients with acute or chronic kidney failure (defined as serum creatinine exceeding 1.5 times the normal value range)
9. Patients with a history of severe allergies or were allergies to metals;
10. Pregnant and lactating women;
11. Poor compliance, unable to follow up on time;
12. Patients participated in other clinical trials within the past three months;
13. The researcher believed that the patients were not suitable for participating in the trial;
14. Patients taking drugs that may affect magnesium metabolism, or organ diseases related to magnesium metabolisms, such as renal, adrenal dysfunction, thyroid, and parathyroid dysfunction

This study used a single-blind method, in which participants were randomly selected to fix the iliac bone flap in one of three ways. The participants were unaware and the researchers did not inform them of the method of fixing the iliac bone flap used.

### Ethical approval status

This prospective, randomized controlled study was registered in Chinese Clinical Trial Registry and approved by the Medical Ethics Committee of the Affiliated Hospital of Nanjing University of Chinese Medicine (2020NL-118-02). Written informed consents were obtained from all participants.

### Surgical methods

Operations were performed by the same surgeon (JRS) who has been engaged in hip preservation for more than three decades. After general anesthesia, the patients were placed in the supine position, and the affected hip elevated 30°. The operation position was selected on the connecting line between the anterior superior iliac spine (ASIS) and the outer edge of the patella, along the iliac bone to 3 cm below the ASIS to the outside of the greater trochanter, and then turned back to the connecting line to form a "S" double curve incision, about 8–12 cm in length. Cut the skin and subcutaneous tissue in turn to protect the lateral femoral cutaneous nerve. One retractor pulled the sartorius muscle and rectus femoris inward, and the other retractor pulled the tensor fascia lata outward to expose the Huter space. Separated from the lower end of the Huter space layer by layer, identified the ascending branch of the lateral circumflex femoral vessels (ALCFVs) and protected it which supplies blood to the separated iliac bone flap with tensor fascia latae). A portion of the tensor fascia latae was dissected from the lateral circumflex femoral artery from its branch near the origin of the lateral circumflex femoral artery until the tensor fascia latae was near the origin of the anterior superior iliac spine. Next, a portion of the ASIS with the origin of the tensor fascia latae (along with the dissociated tensor fascia lata and the ascending branch of the lateral femoral circumflex artery) was harvested. The size of the iliac bone graft was approximately 3*3*1 cm. At the same time, excavate the cancellous bone for iliac. Cut part of the capsule of hip to expose the anterior of the femoral head and neck, chiseled the cartilage which is near to the necrotic area with a grinding drill, then exposed the necrosis bone. Debrided sclerosing bone with a high-speed abrasive drill and debrided necrosis bone with a curet. The necrotic area was filled with the cancellous bone taken out from the iliac bone, and the collapsed femoral head was pressed and enriched with a bone grafting rod. The cancellous bone side of the muscle-bone flap with blood supply was further filled with the necrotic area towards the femoral head, the anterolateral column was rebuilt, and the iliac bone graft was transferred to it. Group A: lifted the collapsed femoral head, measure with a depth meter, selected biodegradable magnesium screws with appropriate diameter and length, drilled it with an electric drill, then drilled out the screw canal with the corresponding tap, finally screwed the biodegradable magnesium screw (35 or 40 mm diameter, Dongguan Eontec.Co., Ltd) to fix the bone graft. Group B: Fixation of the bone graft with a hollow titanium screw (35 or 40 mm diameter, AO Co., Ltd) (as in Group A, without the need to drill a screw channel with a screw tap). Group C: The bone graft was directly embedded without screws. After confirming that the bone graft is firmly fixed, irrigate with normal saline, and close the surgical incision with absorbable line layer by layer, obliterating cavity by stitching tightly. Be careful not to ensnare the lateral femoral cutaneous nerve.

### Postoperative treatment and follow-up

#### Postoperative treatment

Patients were instructed to bend and extend ankle joint, exercise quadriceps femoris, and do gastrocnemius isometric exercises after the operation. Patient began partial weight-bearing walk with the aid of walker 3 months after the operation, and conducted weight-bearing walk without walker 6 months after the operation. The operation times and hospital stays were recorded.

#### Post-operative follow-up

##### Harris scores

The postoperative functional recovery was evaluated through Harris score. The excellent and good rate is the proportion of Harris score above 80 points. 90–100 points are excellent, 80–89 points are good, 70–79 points are medium, and below 69 points are poor.

Functional parameters were assessed separately by one orthopedist and one chief orthopedic physician for each hip of patients who underwent different treatment modalities bilaterally. We evaluated and recorded the operated hip for analysis and excluded the hip joints treated conservatively to minimize errors in the parameters.

##### Radiological evaluations

1. X-ray: X-ray(orthopantomogram and frog-mogram) was taken on 1, 3, 6 months after surgery to assess the preoperative morphological location of necrotic areas, postoperative morphology, progression of ONFH and the amount of gas production of the magnesium screws.
2. CT: The femoral head was evaluated by CT coronal measurements on postoperative day 1, 3 and 6 months postoperatively. CT was used to evaluate specifically the postoperative iliac bone graft position, the loosening of the magnesium screw, the amount of gas production, the growth and fusion of the bone graft with the surrounding normal tissue, and the collapse of the femoral head.
3. DCE-MRI: All MRI experiments were performed using a 3.0 T MRI (SIGNA Architect) with a air coil. The "double line sign" of femoral head necrosis was selected as the boundary according to the T2-weighted MRI image, with the necrotic zone on the medial side and the repair response zone between the "double lines". Coronal DISCO (Differential Sub-sampling with Cartesian Ordering) imaging was performed with a field of view of 36 × 36 cm, covering the area of entire pelvis and proximal femur. The other scan parameters included a repetition time = 4.2 ms, echotime = 1.5 ms, a flip angle = 12°, a slice thickness = 1.0 mm, and a matrix size = 360 × 360. Before DISCO imaging, gadoteric acid meglumine salt (0.4 mL/kg) was injected as the contrast agent at a rate of 3 mL/s. Coronal DISCO imaging with a total 15 arterial phases was performed 10 s after the onset of contrast agent administration. Each single-period scan took 12.7 s; therefore, the total scan time was 192 s. Datum was processed using GE's Geniq software. Regions of interest (ROI) were plotted in necrotic area, the repaired area, and the greater trochanter, and time-signal intensity curves (TIC) were obtained and classified for all pixel points within the region of interest. The area under the curve (IAUGC), peak contrast-enhanced signal (CER), maximum slope of the curve (MaxSlope), volume shift constant (Ktrans), volume rate constant (Kep), and extracellular interstitial volume fraction outside the vessel (Ve) were calculated. IAUGC reflects whole blood flow, CER reflects capillary density, MaxSlope reflects blood flow velocity, and Ktrans and Kep reflect nutrient flow. Ktrans and Kep reflect nutrient exchange capacity, and Ve reflects tissue oedema. The reconstruction and recovery of blood supply of the femoral head were evaluated and the microenviromental blood supply changes were observed.

A senior radiologist with at least 5 years of clinical experience first independently reviews all of the patient's pre- and post-operative imaging data. Subsequently, the radiologist conducts radiological evaluations together with one orthopedist and one chief orthopedic physician, to ensure accuracy and consistency in the interpretation of the imaging data.

##### Adverse post-operative complications

We conducted post-operative follow-up to detect infection and non-healing of the incision. We evaluated serum magnesium ion level according to liver and kidney function, detect the deep veins of both lower limbs by ultrasound, detect subcutaneous emphysema and fracture of the magnesium screw by X-ray and CT.

### Efficacy evaluation indicators

(1) success rate of surgery; (2) radiological evaluation of the location of bone graft at 3 and 6 months after surgery; (3) Harris score of the patient before and 6 months after surgery; (4) femoral head collapse rate at 6 months after surgery; (5) observation of bone ingrowth by CT at 6 months after surgery; (6) observation of changes in microcirculation of the femoral head by DCE-MRI before and 6 months after surgery.

### Statistical methods

SPSS 26.0 software was used for statistic analysis. The χ2 test or Fisher's exact probability method was used for comparison among three groups. One-way ANOVA was used for the comparison among groups, and independent samples *t*-test was used for further comparison between groups of non-normally distributed measures. The measures were tested to be normally distributed at *α* = 0.05.

## Results

### General information

A total of 36 patients met the selection criteria for inclusion in the study from March 2021 to July 2022. Three patients (3 hips) had mild liver function abnormalities during the course of the study. We reported this phenomenon to ethics committee, and they deemed it to have no serious impact on the trial. The 36 patients were divided into a trial group and two control groups. Biodegradable magnesium screws were implanted during PVIBGT in 12 patients (12 hips) in Group A. Common screws were implanted during PVIBGT in Group B (12 patients, 13hips). Iliac bone grafts were directly embedded during PVIBGT in Group C(12 patients, 12 hips).

Group A consisted of 12 patients with a total of 12 affected hips, including 10 males (10 hips) and 2 females (2 hips) with an age range of 27–48 years with a mean age of 33 years. Of the 12 hips, 6 were ARCO stage II and 6 were stage III. The ONFH types were as follows: 3 cases of hormonal ONFH involving 3 hips, 3 cases of alcoholic ONFH involving 3 hips, 1 case of traumatic ONFH involving 1 hip, and 5 cases of idiopathic ONFH involving 5 hips. The duration of ONFH ranged from 1 to 25 months with a mean of 10 months.

Group B included 12 patients with a total of 13 affected hips, consisting of 6 males (7 hips) and 6 females (6 hips). The age range was 18–43 years old with an average age of 29.9 years. Of the 13 hips, 6 were ARCO stage II and 5 were stage III. The ONFH types were as follows: 7 cases of hormonal ONFH involving 8 hips, 1 case of traumatic ONFH involving 1 hip, and 4 cases of idiopathic ONFH involving 4 hips. The duration of ONFH ranged from 1 to 25 months with a mean of 11 months.

Group C comprised 12 patients with a total of 12 affected hips, including 10 males (10 hips) and 2 females (2 hips). The age range was 21–40 years old with a mean age of 31.9 years. The distribution of ARCO stage was 7 stage II and 5 stage III. The ONFH types were as follows: 3 cases of hormonal ONFH involving 3 hips, 5 cases of alcoholic ONFH involving 4 hips, and 4 cases of idiopathic ONFH involving 4 hips. The duration of ONFH ranged from 1 to 24 months with a mean of 6 months.

Among the 36 subjects in our study, 19 patients had bilateral femoral head necrosis. Only one patient underwent bilateral PVIBGT due to the severity of their condition, significant pain, and strong demand for surgery. There maining 18 subjects underwent unilateral PVIBGT. For the 18hip joints with bilateral femoral head necrosis that did not undergo PVIBG, we adopted conservative treatment based on their conditions: avoid weight-bearing for three months.

The differences were not statistically significant (*P* > 0.05) in gender, age, duration of disease, side, type and stage of ONFH among three groups. More details are shown in Table 1.

**Table 1:** Comparison of clinical data of the three groups of patients

Patient information	Group A	Group B	Group C
Gender (M/F)	10/2	6/6	10/2
Average age (years)	33.5	29.9	31.9
Average surgery time (min)	175.4	187.4	191.6
Average length of stay (d)	18.2	18.12	8.1
Type of necrosis (Hormonal/Alcoholic/Traumatic/Idiopathic)	3/3/1/5	7/0/1/4	3/5/0/4
Unilateral/Bilateral Surgery	12/0	11/1	12/0

There were no statistic difference among Group A, B and C

### Comparison of the operation time and hospital stay of patients in the three groups

All three groups of patients had successful surgery. The average operating time per side of the hip was 178.18, 179.42 and 183.18 min in Group A, B and C, respectively, and the patients' hospital stay was 18.18, 18.12 and 18.09 d, respectively, with no statistically significant difference among three groups (*P* > 0.05).

### Harris score

**Fig. 2:**
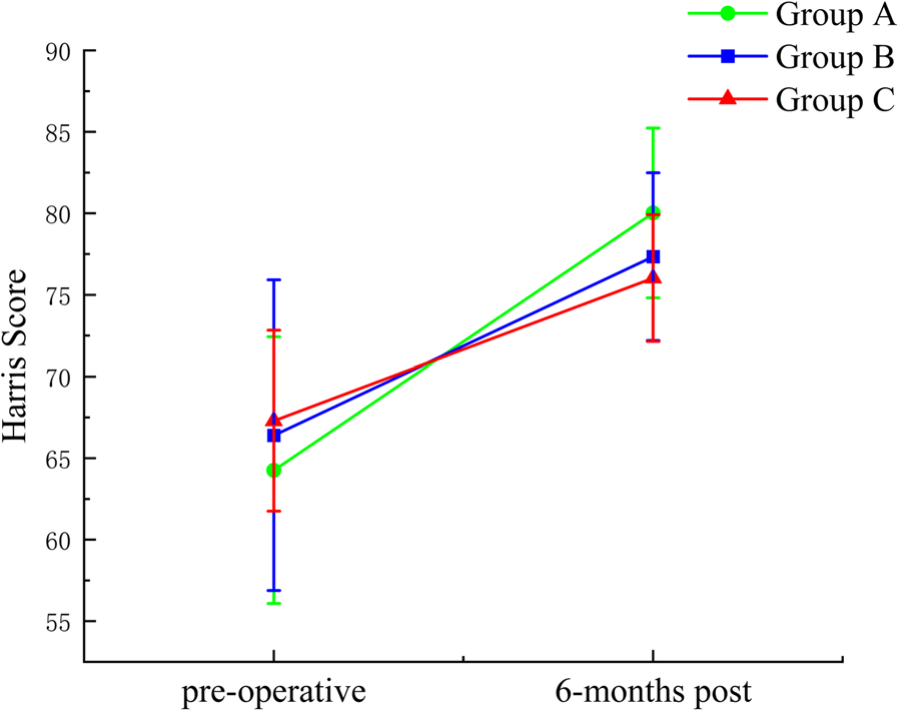
Changes of mean Harris score of three groups preoperatively and 6 months postoperatively.Harris scores improved significantly in all 3 groups, with the most significant improvement in Group A (green)

Group A had a mean Harris score of 64.26 preoperatively and 80.03 at 6 months postoperatively. Group B had a mean Harris score of 66.40 preoperatively and 77.34 at 6 months postoperatively. Group C had a mean Harris score of 67.29 preoperatively and 76.03 at 6 months postoperatively. All three groups showed a statistically significant (*P* < 0.05) difference in preoperative and postoperative Harris scores. At 6 months of follow-up, there are 1 excellent hips, 7 good hips and 0 poor hips in Group A; 0 excellent hips, 6 good hips and 0 poor hips in Group B; 0 excellent hips, 4 good hips and 0 poor hips in Group C. The excellent and good rates in Group A, B, C were 66.7%, 46.2%, 33.3%, respectively. More details are shown in Table 2 and Fig. 2.

**Table 2:** Comparison of Harris scores between three groups before and after surgery

Group	Number of cases	Harris socre		*D*-value
		Preoperative	6 months-post	
A	12 (12 hips)	64.26 ± 8.17	80.03 ± 5.21^△^	15.78 ± 8.73^*^
B	12 (13 hips)	66.40 ± 9.52	77.34 ± 5.15^△^	10.94 ± 7.64
C	12 (12 hips)	67.29 ± 5.54	76.03 ± 3.89^△^	8.74 ± 6.18

All data were in accordance with normal distribution. The preoperative and postoperative differences were compared by one-way ANOVA, and multiple comparisons were made by LSD method; The Harris scores were compared before and 6 months after surgery using paired *t*-tests. ^△^Compared with preoperative results, *P* < 0.01;* Compared with the other two groups, *P* < 0.01

### Radiological results and evaluation

#### Typical patients in three groups

**Fig. 3:**
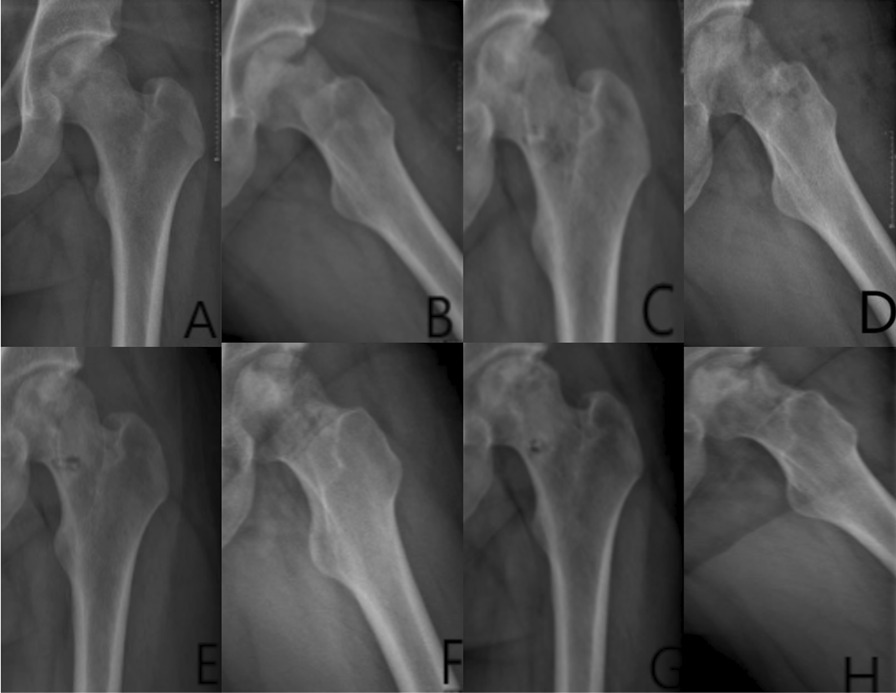
Pre-operative (**A**, **B**), 1-day (**C**, **D**), 3-month (**E**, **F**), 6-month post-operative (**G**, **H**) X-rays (ortho and frog) of a typical patient in Group A. CT and 3D reconstruction

**Fig. 4:**
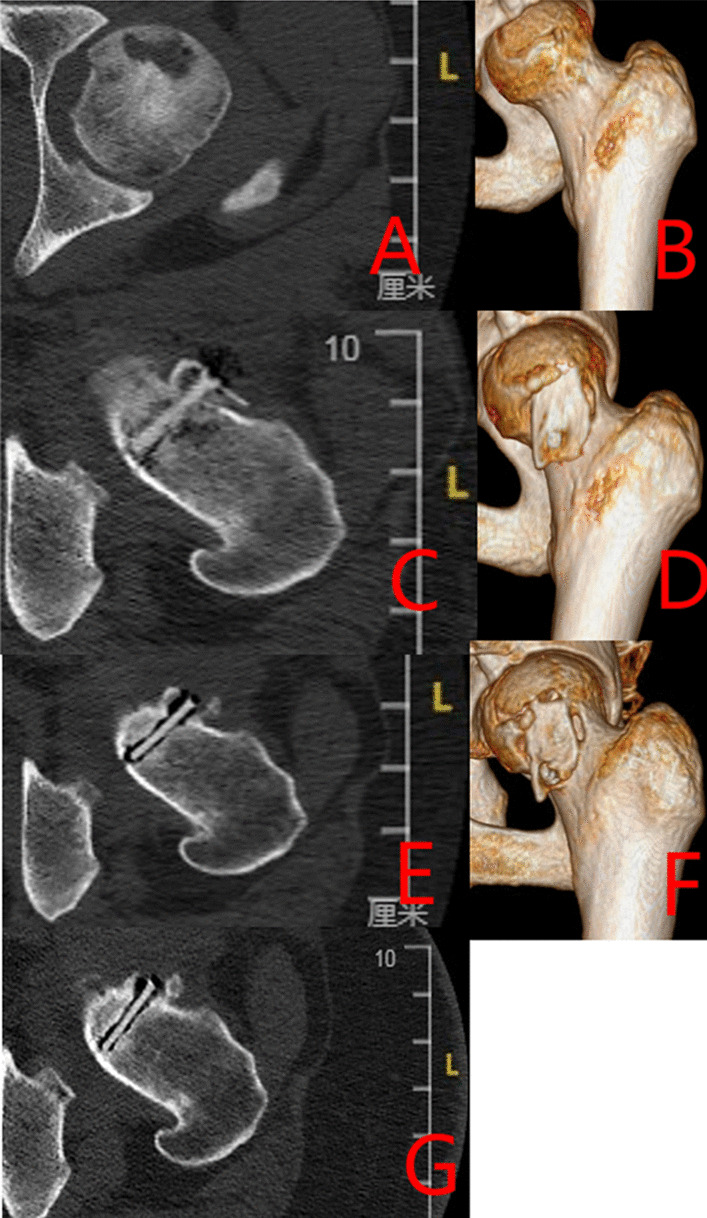
Pre-operative (**A**, **B**), post-operative (**C**, **D**), 3-month (**E**–**F**) and 6-month (**G**) CT and 3D reconstructions of a typical patient in Group A. The magnesium screw used to fix the graft bone flap is visible. The degradation of the magnesium screw can be seen in the picture, with the bone flap firmly in position and the growth of bone around the screw, which also demonstrate the osteogenesis of the screw
- Group B: The patient has bilateral hormonal ONFH.The patient underwent bilateral PVIBGTs with two titanium screws. More details are shown in Figs. 5 and 6.

- Group A: The patient has left-sided hormonal ONFH. The patient’s hip was fixed with a biodegradable magnesium screw (4.0 mm diameter, 30 mm long) in the PVIBGT. More details are shown in Figs. 3 and 4.

**Fig. 5:**
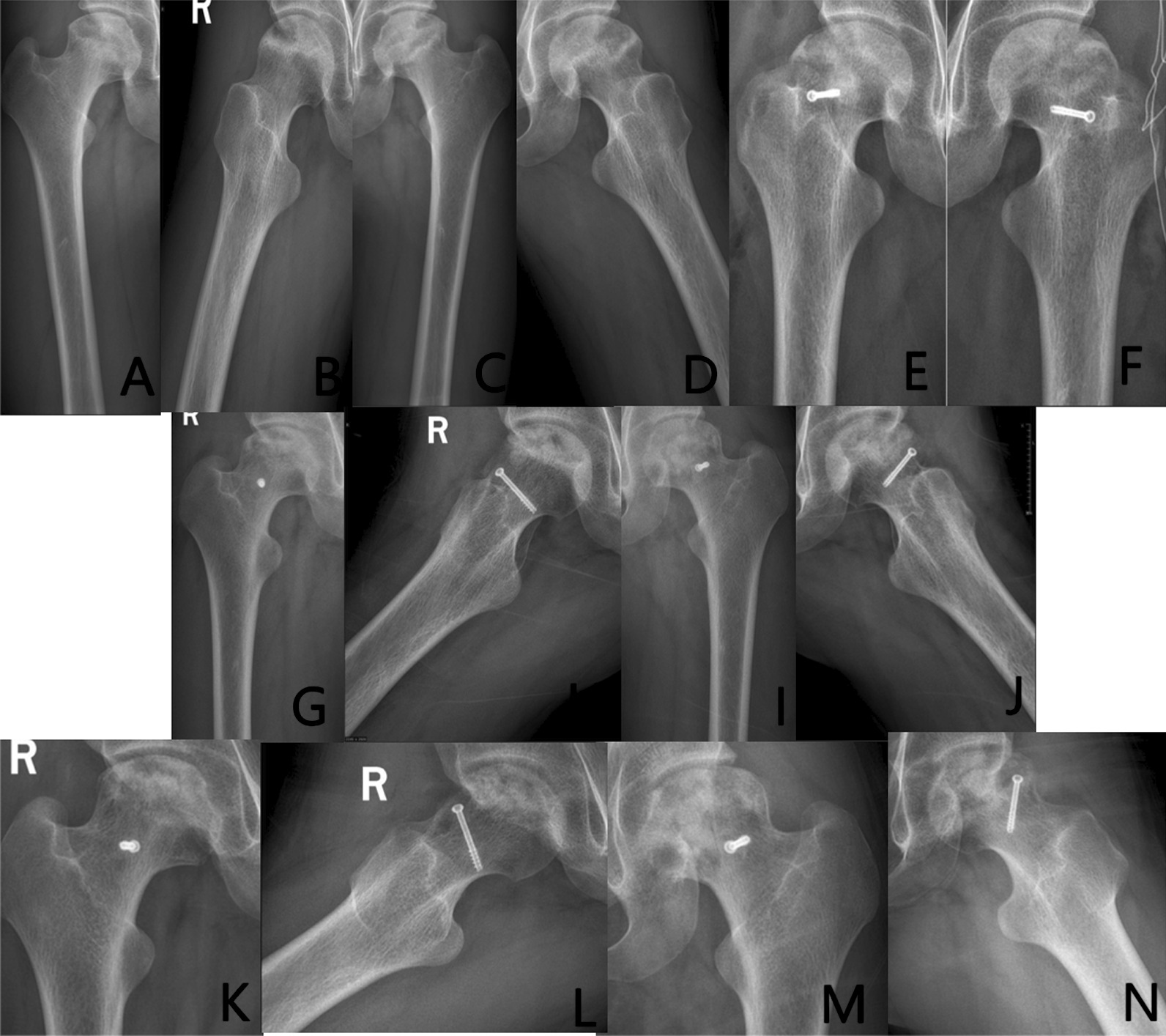
Pre-operative (**A**–**D**), 1-day post-operative (**E**–**F**), 3-month post-operative (**G**–**J**) and 6-month post- operative (**K**–**N**) X-rays (ortho and frog) of a typical patient in Group B

**Fig. 6:**
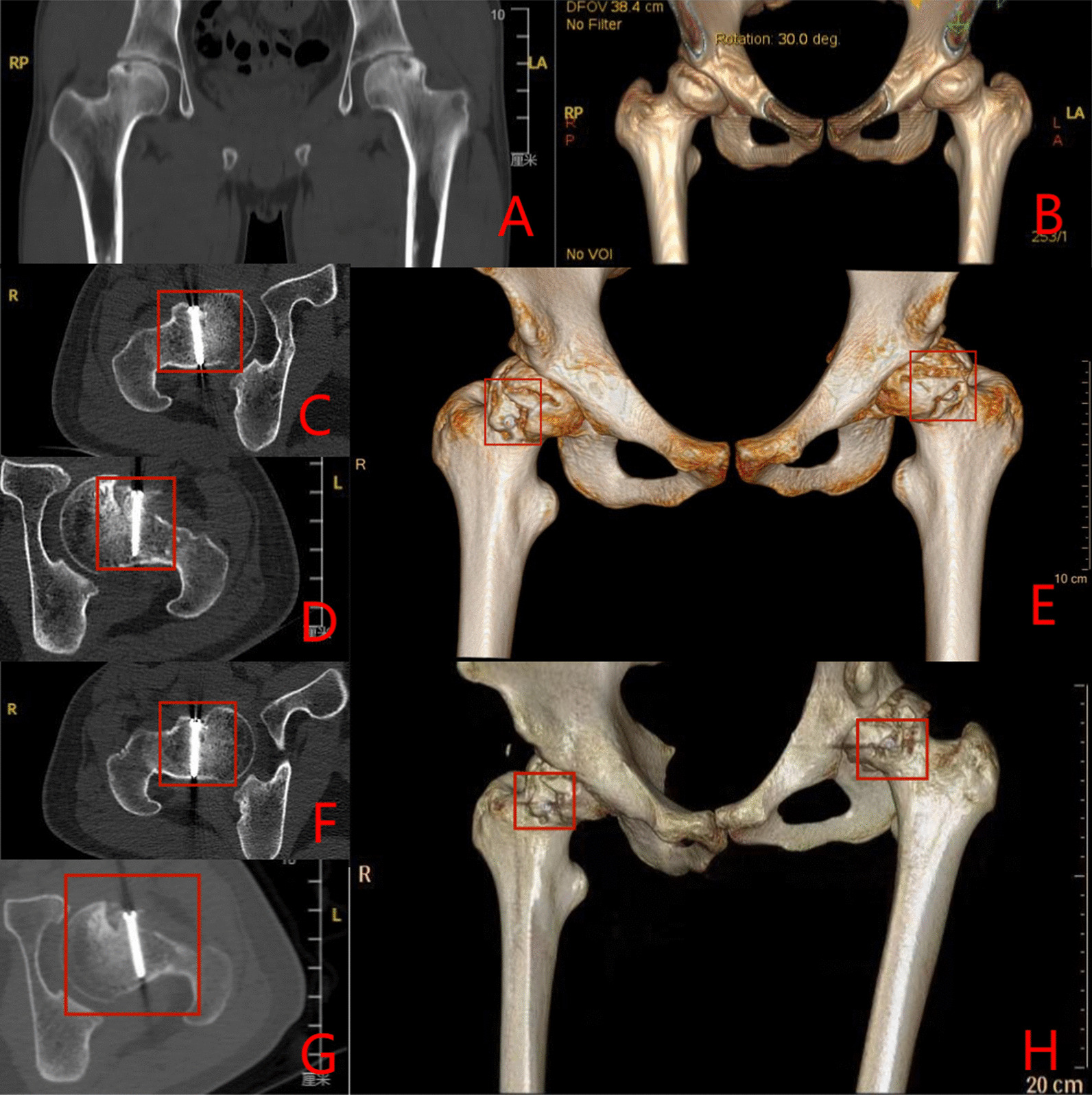
CT and 3D reconstruction of a typical patient in Group B before surgery (**A**–**B**), 3- months (**C**–**E**) and 6-months after surgery (**F**–**H**). The titanium screws used to fix the graft bone flap is visible (red box)
- Group C: The patient has left idiopathic ONFH. The patient underwent a left-sided PVIBGT without any screw (directly embedded iliac bone graft). More details are shown in Figs. 7 and 8.

**Fig. 7:**
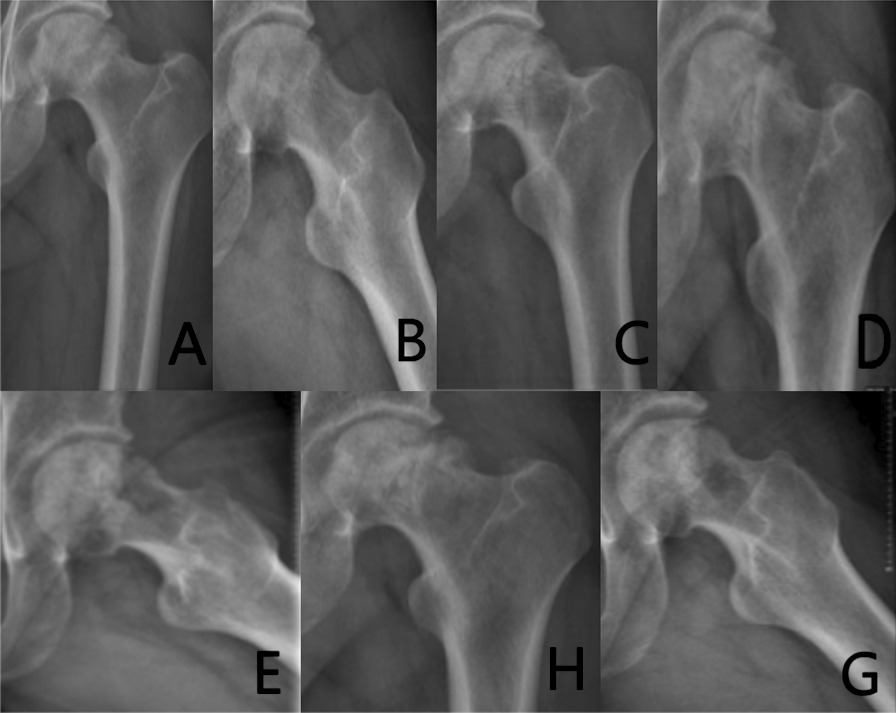
Pre-operative (**A**, **B**), post-operative (**C**), 3-month post-operative (**D**, **E**) and 6-month post-operative (**F**, **G**) X-rays (ortho and frog) of a typical patient in Group C

**Fig. 8:**
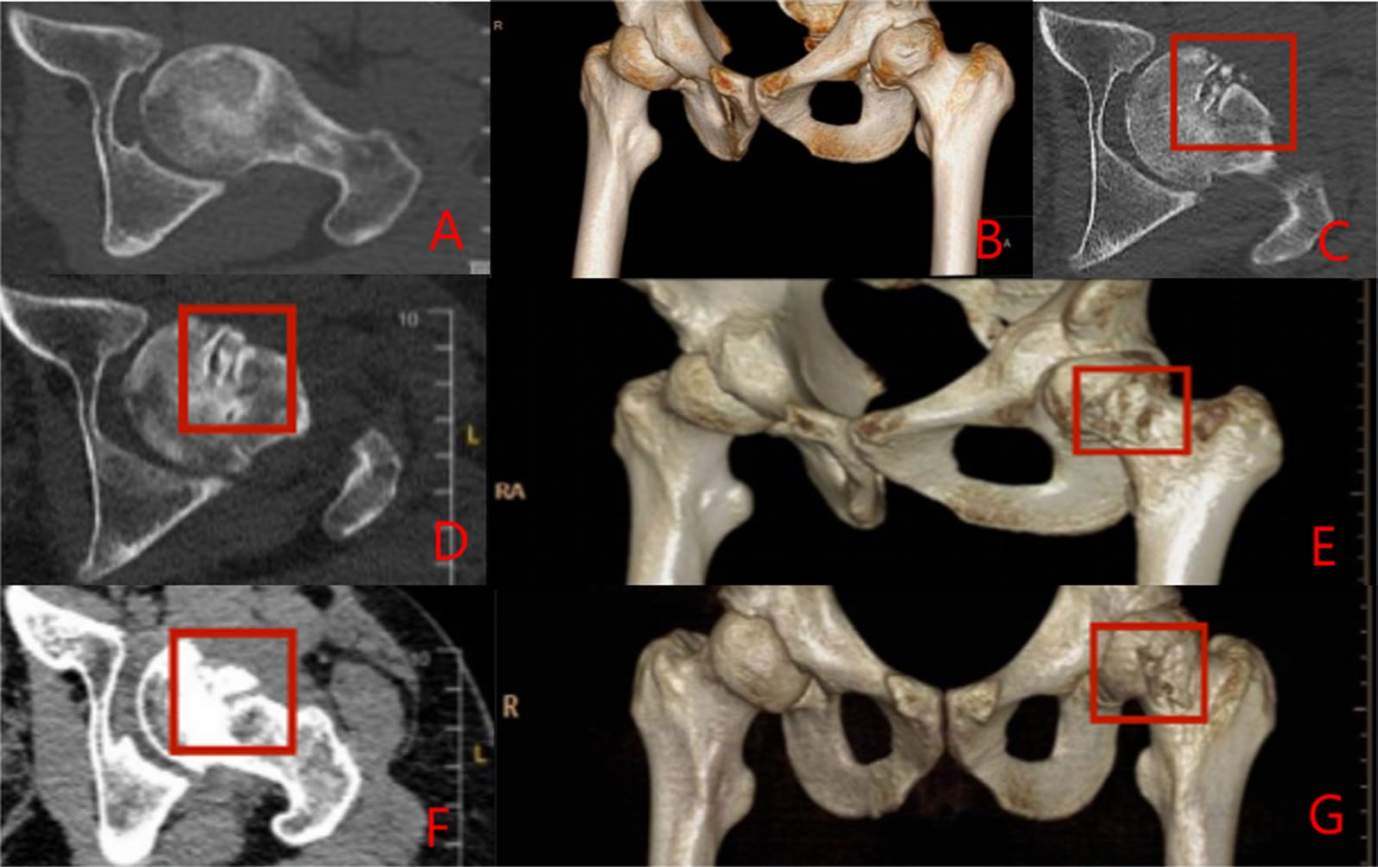
CT and 3D reconstruction of a typical patient in Group C before surgery (**A**, **B**), 1-day post-operative (**C**), 3 months after (**D**, **E**) and 6 months after surgery (**F**, **G**).The grafted bone flap is in red box

#### Evaluation blood supply via DCE-MRI

**Fig. 9:**
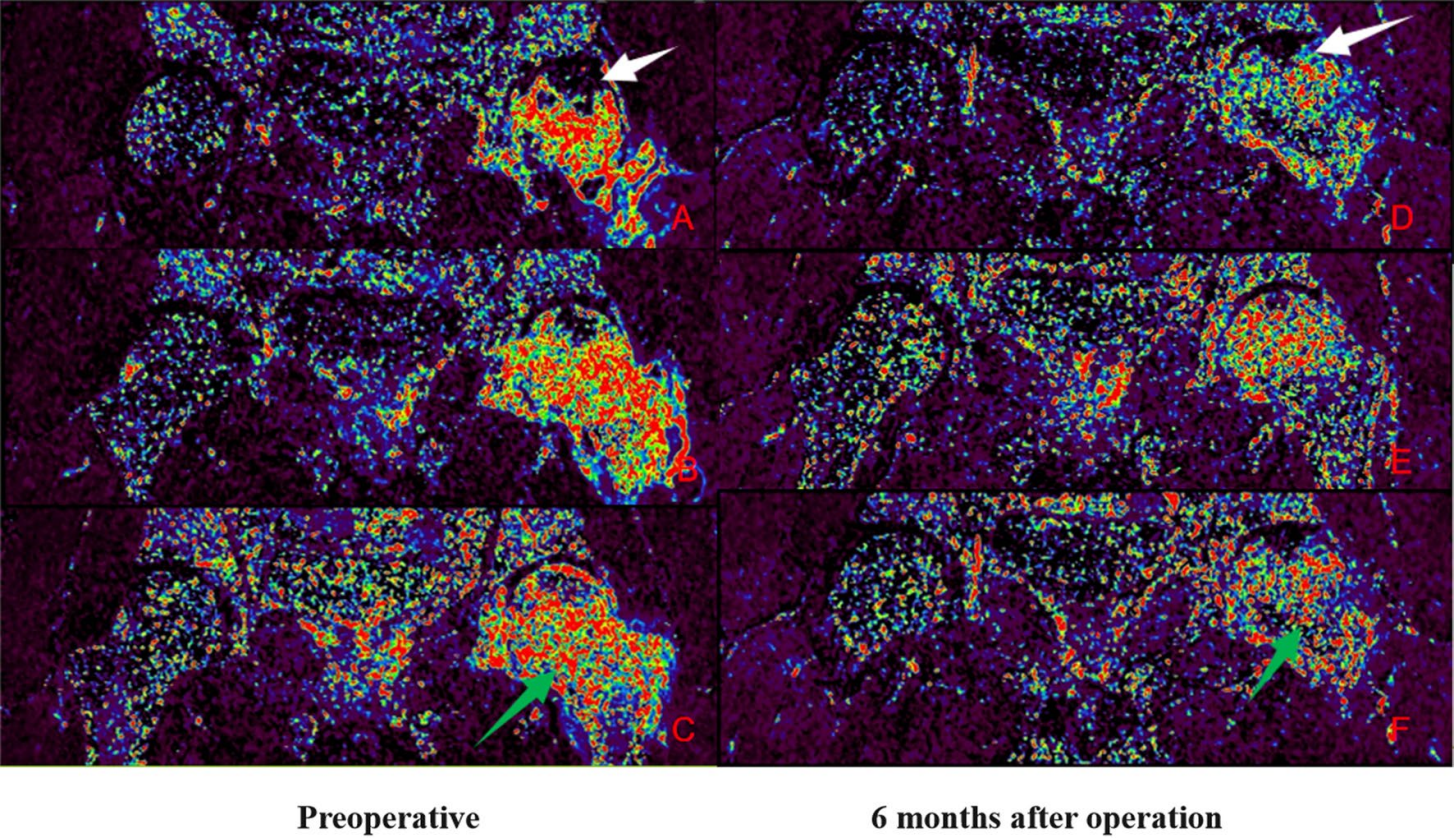
IAUGC and Ktrans synthetic pseudo-colour images. At 6 months postoperatively, there was a significant reduction in abnormal hyperperfusion in the repaired area (green arrow) and a significant improvement in microcirculation in the necrotic area (white arrow) compared to the preoperative period

**Fig. 10:**
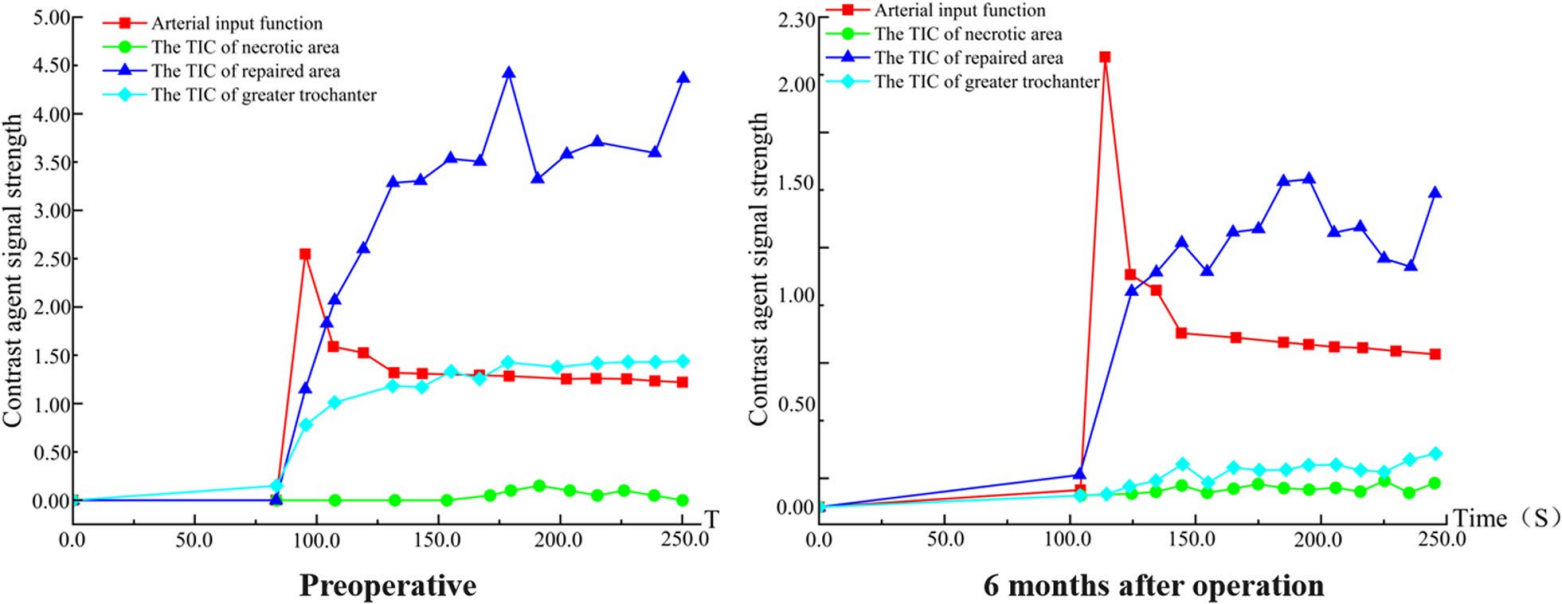
Time-intensity curve (TIC). The perfusion of necrotic area increased and peak of perfusion decreased in the repair area 6 months after surgery. This suggests an improvement in microcirculation in the necrotic area and a decrease in abnormal hyperperfusion in the repair response area. The microcirculatory perfusion in the necrotic and repaired areas gradually converged to normal bone tissue
- Group B: Patient Lu XX, male, 21 years old, with bilateral hormonal ONFH. The patient underwent bilateral PVIBGT with magnesium screws. More details are shown in Figs. 11 and 12.

- Group A: Patient Yan X, male, 29 years old, with left-sided hormonal ONFH. The patient’s hip was fixed with a biodegradable magnesium screw. More details are shown in Figs. 9 and 10.

**Fig. 11:**
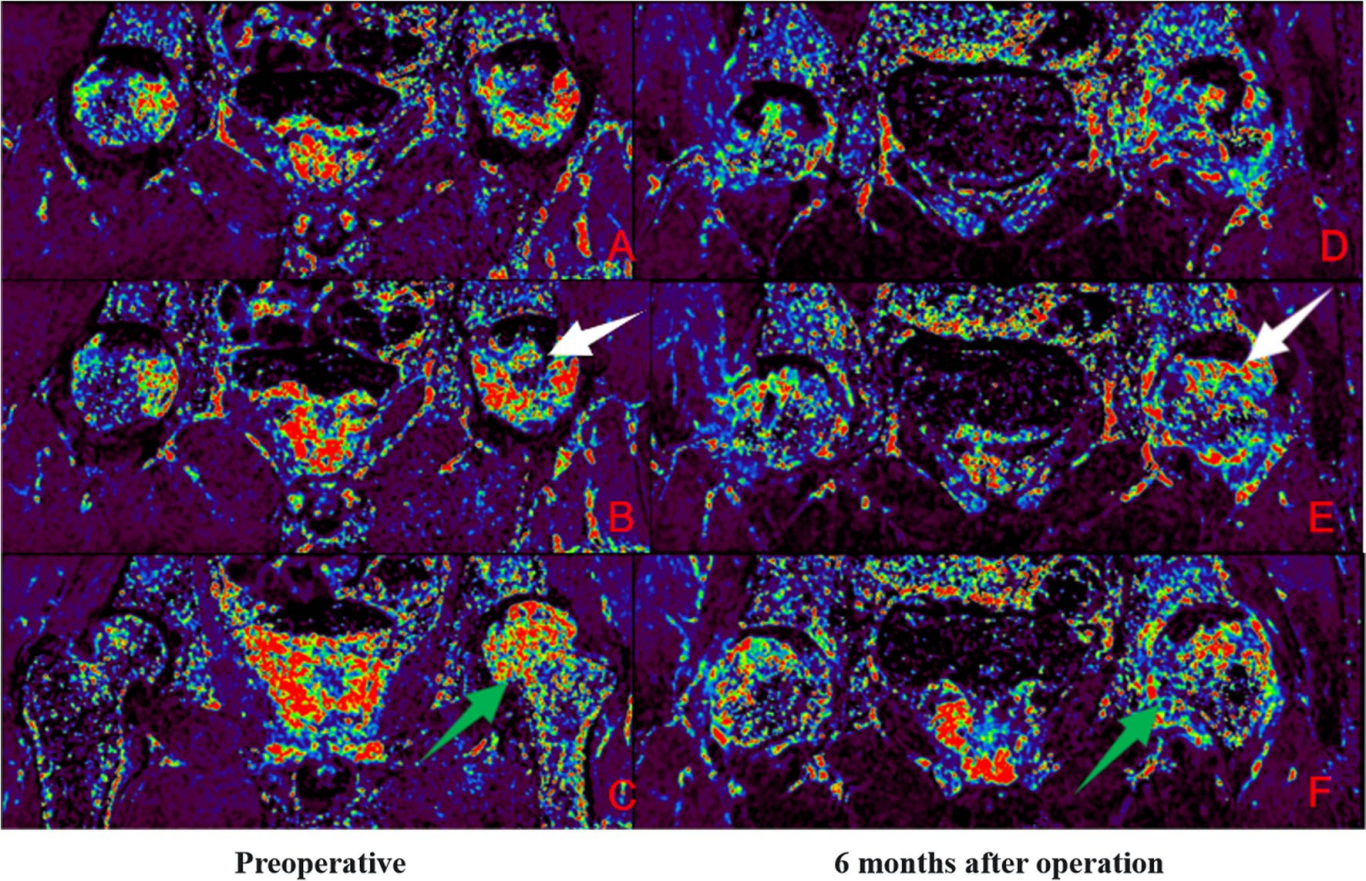
IAUGC and Ktrans synthetic pseudo-color images. Compared to the preoperative period, abnormal hyperperfusion in the repaired area was significantly reduced at 6 months postoperatively (green arrow), but microcirculation in the necrotic area was not as improved as in Fig. 9 (white arrow)

**Fig. 12:**
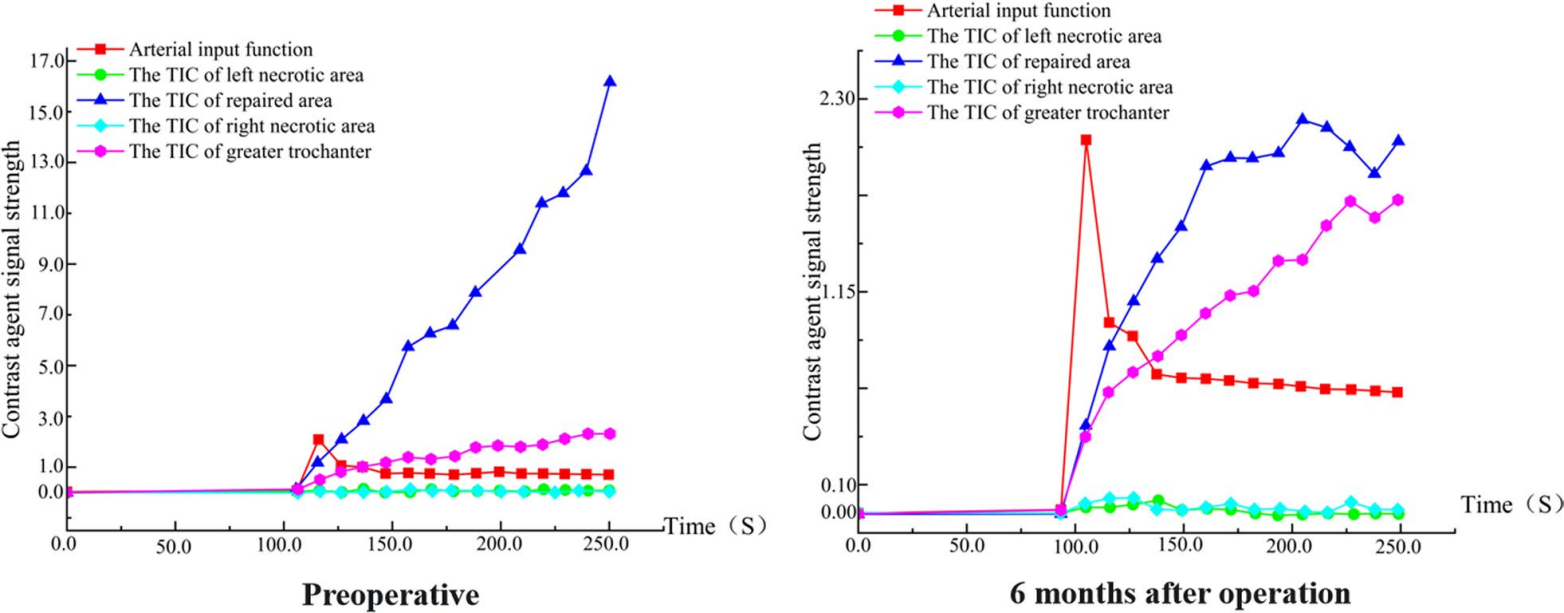
Time-intensity curve (TIC). The perfusion increased in necrotic area and peak of perfusion decreased in the repair area 6 months after surgery. A decrease in abnormal hyperperfusion in the repair-responsive area can be clearly observed in the figure, but the improvement in microcirculation in the necrotic area is less pronounced than in group A (Fig. 10), which also demonstrates the improved effect of the biodegradable magnesium screw on blood supply
- Group C: Patient Peng XX, male, 33 years old, with left idiopathic ONFH. The patient underwent a left-sided PVIBGT without any screw. More details are shown in Figs. 13 and 14.

**Fig. 13:**
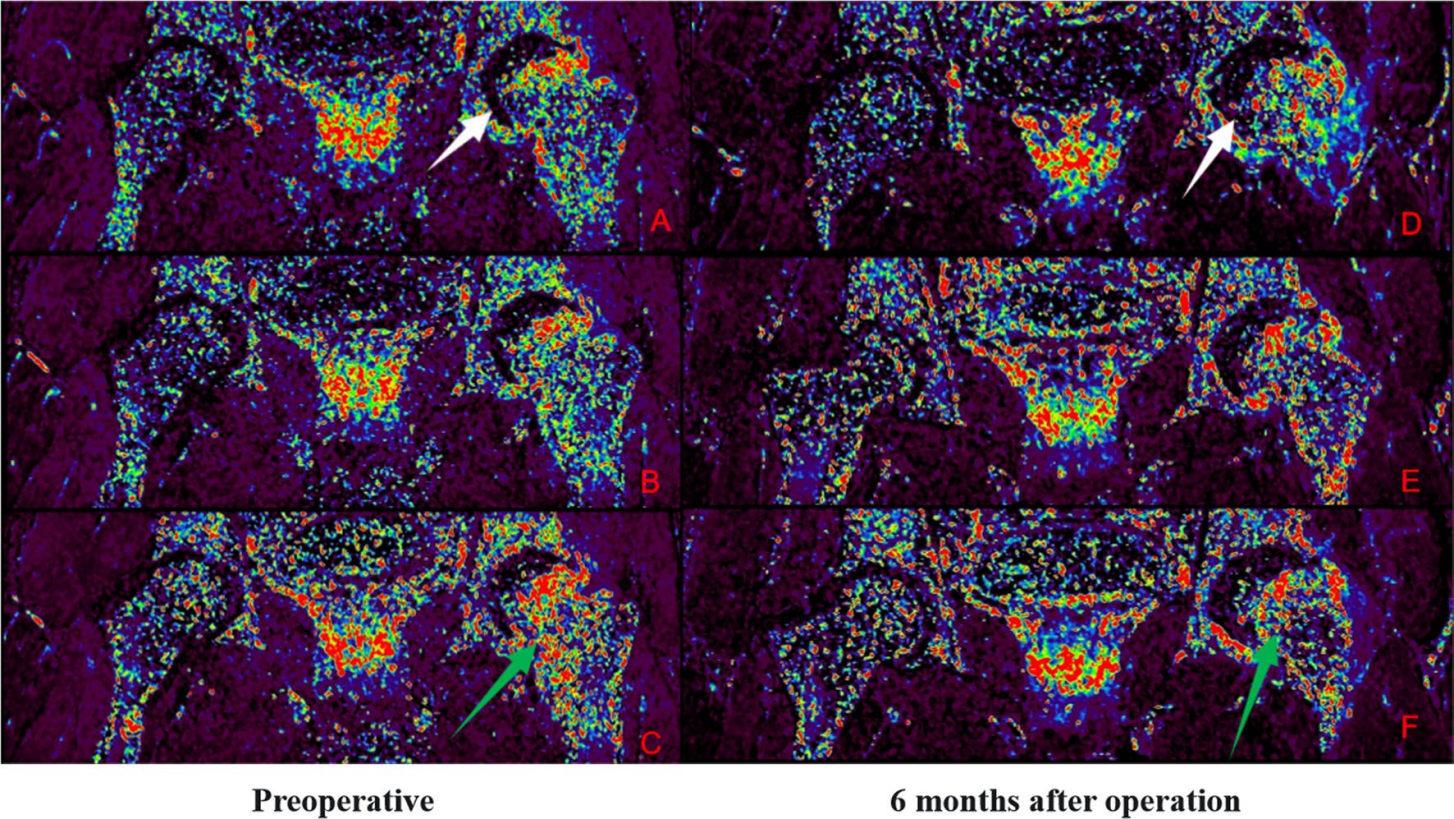
IAUGC and Ktrans synthetic pseudo-color images. Compared to the preoperative period, at 6 months postoperatively, there was a significant reduction in abnormal hyperperfusion in the repaired area (green arrow), but no significant improvement in microcirculation in the necrotic area (white arrow)

**Fig. 14:**
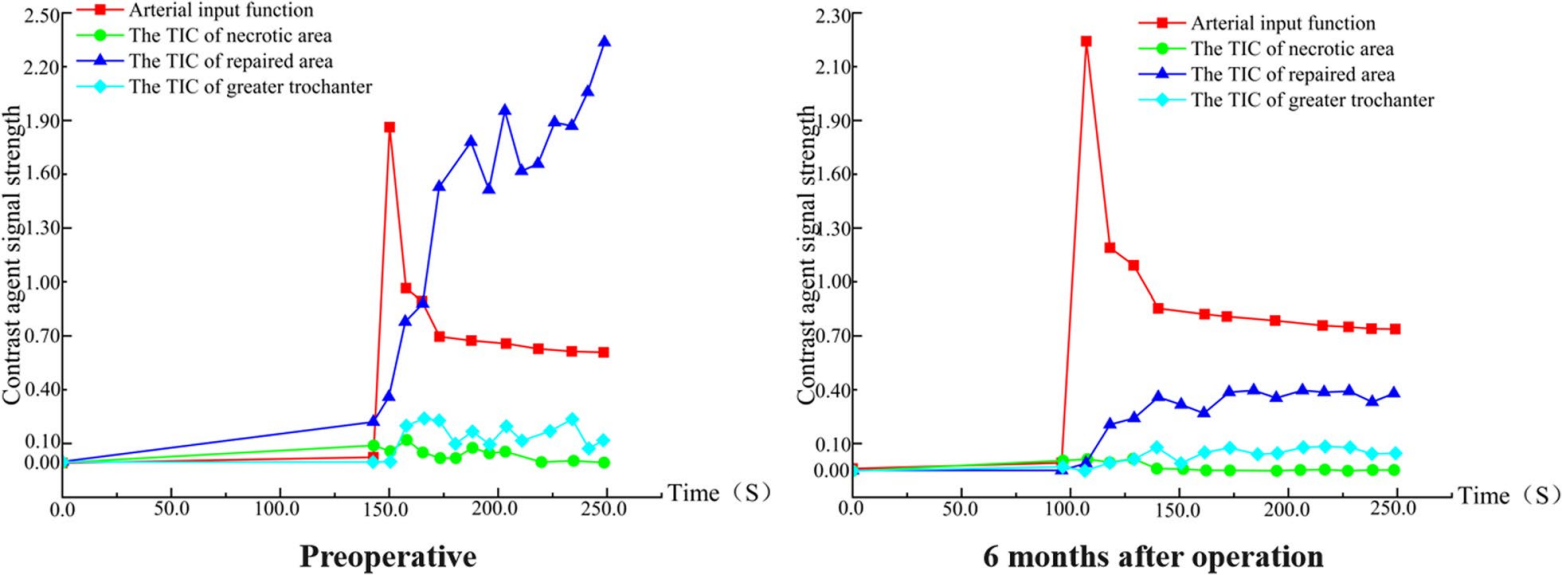
Time-intensity curve (TIC). The perfusion increased in necrotic area and peak of perfusion decreased in repaired area 6 months after surgery. The tendency of microcirculation in the repair-responsive area to normal bone tissue can be clearly observed in the figure, but the improvement in microcirculation in the necrotic area is also less pronounced than in group A (Fig. 10)

#### Degradation rate and air production of biodegradable magnesium bone internal fixation screws

**Fig. 15:**
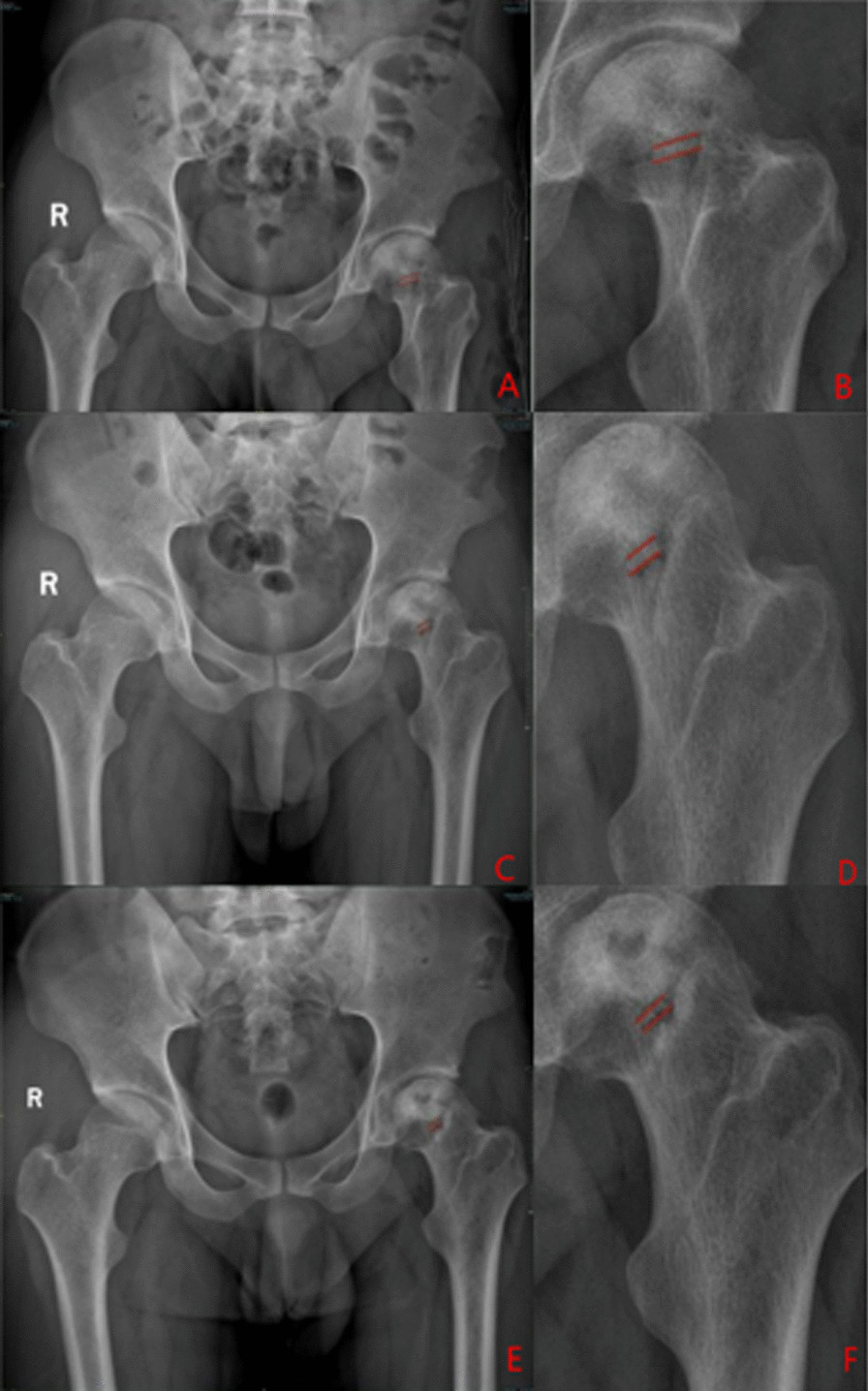
Post-operative (**A**, **B**), 3-month (**C**, **D**) and 6-month post-operative (**E**, **F**) radiographs of a typical patient showing changes in the size of biodegradable magnesium screw

**Fig. 16:**
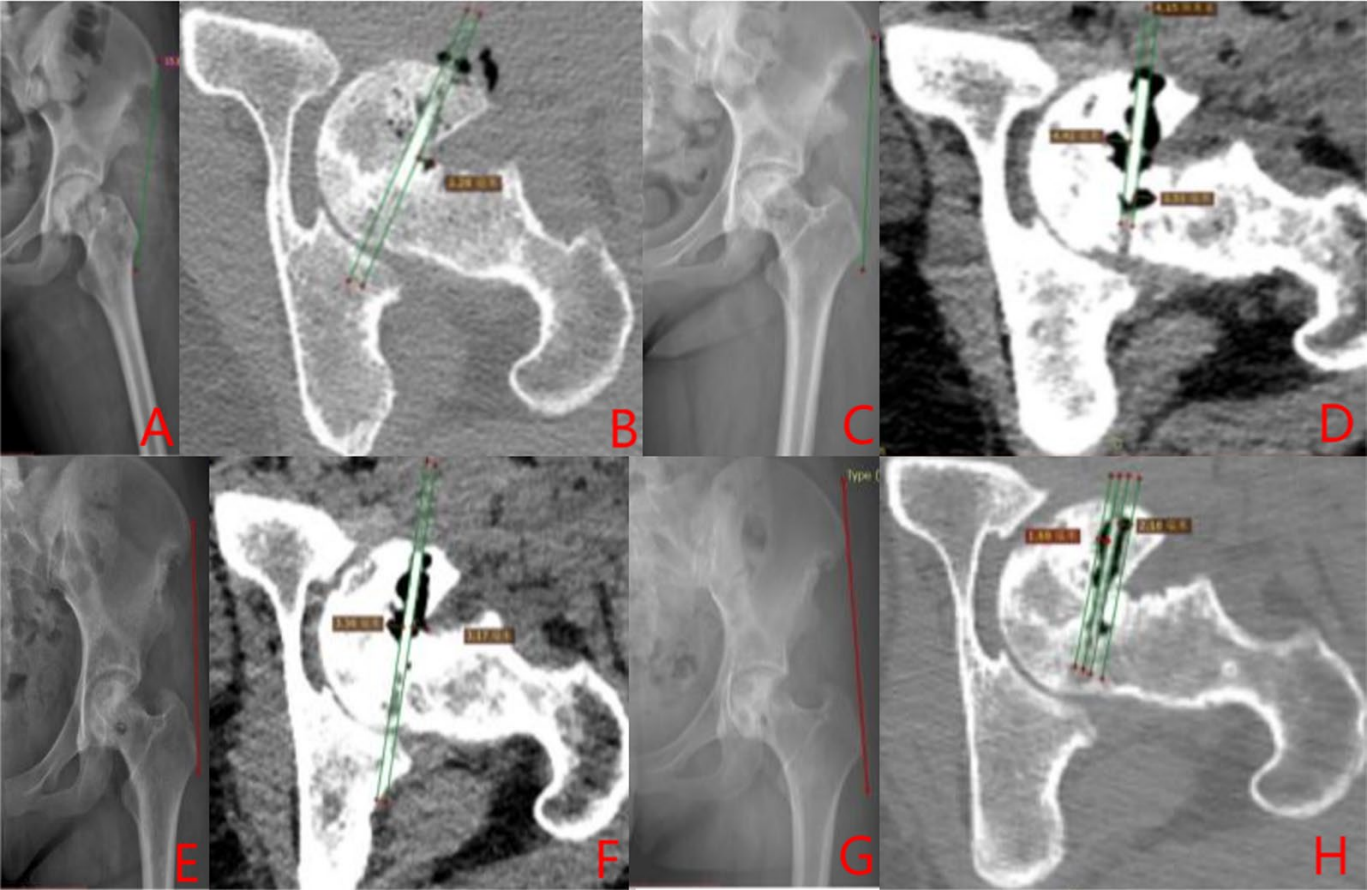
Typical patients in Group A were followed up at 14 days (**A**, **B**), 4 weeks (**C**, **D**), 3 months (**E**, **F**) and 6 months after surgery (**G**, **H**): the area of the interosseous air cavity on X-ray orthopantomography was 0 cm^2^, the distance between the outer edge of the intraosseous air cavity and the edge of the screw varied from 0.65–0.66 to 0.93–0.84 cm on CT

1. The degradation rate of biodegradable magnesium screws was approximately 10.32% at 3 months and 13.72% at 6 months postoperatively in Group A (Fig. 15).
2. The area of the intermuscular air space of the biodegradable magnesium screw was 0 cm^2^. More details and CT results are shown in Fig. 16.

### Analysis of serum biochemical indexes

**Fig. 17:**
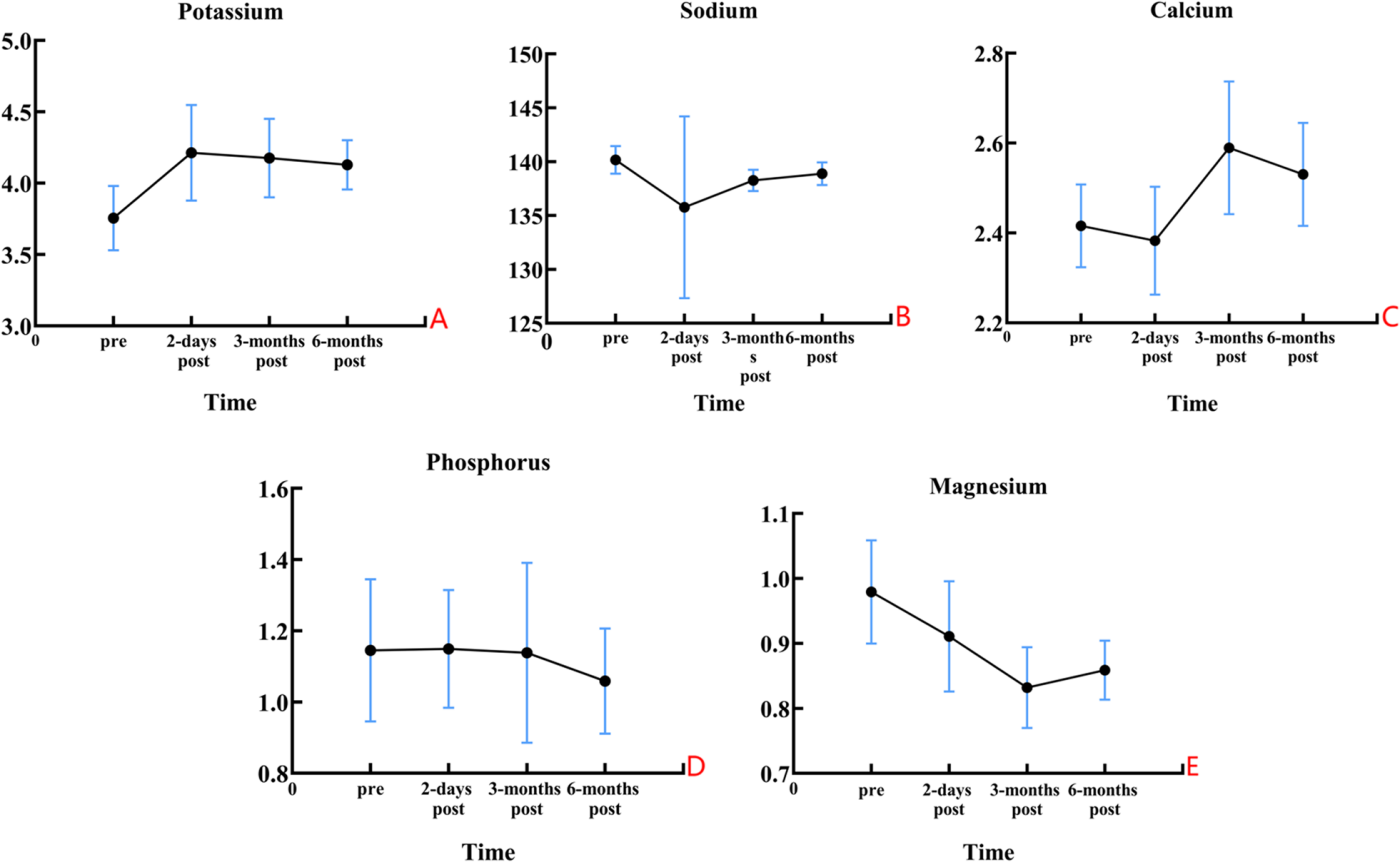
Average serum potassium, sodium, calcium, phosphorus and magnesium ion levels in Group A patients preoperatively, 2-days postoperatively, 3-months postoperatively and 6-months postoperatively. Therefore, the postoperative serum metal ions did not change significantly in Group A patients compared to the preoperative period due to the implantation of the biodegradable magnesium screw, which also proved the safety of the biodegradable magnesium screw

The levels of serum potassium, sodium, calcium, phosphorus and magnesium ion were in normal range, and other indexes of kidney function were normal in Group A. More details are shown in Fig. 17.

### Adverse reactions

**Fig. 18:**
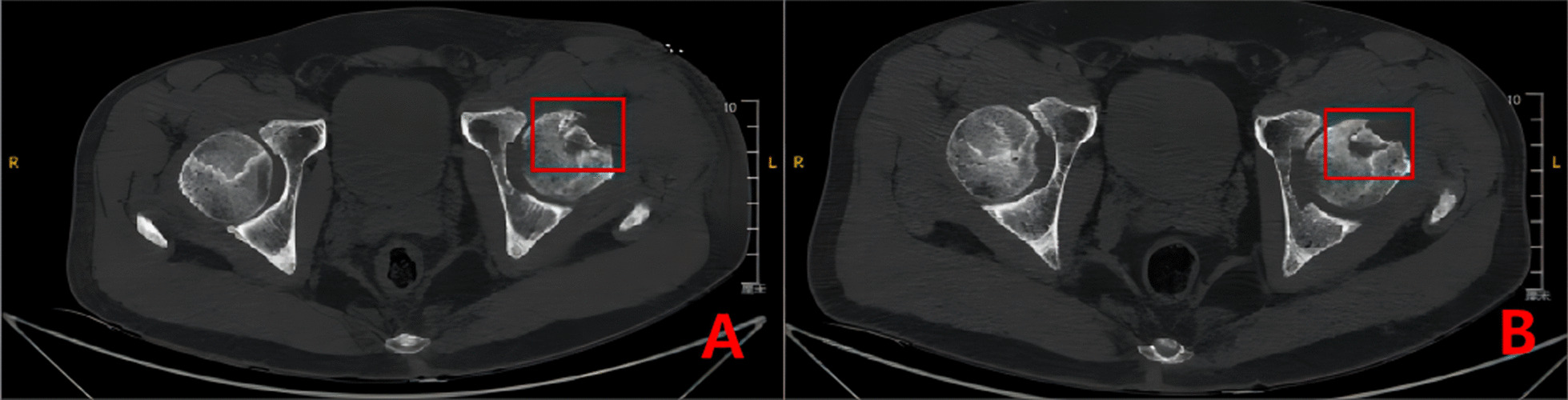
Postoperative (**A**) and 3-month postoperative (**B**) CT of patient Wang X in Group C. Mild displacement of the implanted bone flap can be seen (red box)

While two patients in Group A had poor healing of the surgical incision, other patients in Group A did not have any hip infection, non-healing incision, deep vein thrombosis in the lower limbs, or other adverse events. Group A had good biocompatibility and no fractures of the magnesium screws. Two patients with poorly healed surgical incisions were treated promptly and eventually recovered well. More details are shown in Fig. 18.

## Discussion

### Advantages of PVIBGT in hip preservation

There are 8.12 million ONFH patients in China [12]. The main pathogenesis is the blood supply of the femoral head impaired. The commonly used femoral head-preserving procedures in clinical practice include transtrochanteric rotational osteotomy, core decompression, and PVIBGT, etc [13–15]. PVIBGT is an effective method to ARCO stage II and III ONFH [16, 17]. In this study, the Harris score improved significantly in all groups(compared with preoperative Harris score at 6 months after surgery, the average improvement in Group A was about 15.78, Group B was about 10.94, and Group C was about 8.74), indicating the excellent clinical outcome of the PVIBGT. Taking advantage of both biological and mechanical repairs, this surgical method is able to deal with the mechanical support of the necrotic area and the recovery of blood supply at the same time [18].

### Advantages of biodegradable magnesium screw-fixed bone flaps

#### Biodegradable magnesium screw promotes osteogenesis and angiogenesis

Mg^2+^ has a significant osteogenic effect in vivo and vitro [19]. On the one hand, Mg^2+^ enhances the release of calcitonin-gene-related peptide (CGRP) from sensory nerve endings in periosteal tissues and promotes osteogenesis. Zhang Yifeng [20] et al. found that Mg^2+^ generated from the degradation of magnesium needles could diffuse to the vicinity of the periosteum, enhancing the release of increased sensory nerve endings in the periosteal tissues of rats with femoral fractures, which in turn promoted osteogenesis. Qin Ling [21] developed a magnesium containing hybrid intramedullary nail fixation system (Mg-IMN) to promote the healing of atypical femoral fractures (AFF) by enhancing the synthesis and release of CGRP. On the other hand, Mg^2+^ promotes osteogenesis by promoting inflammatory immune microenvironment in the early stages of osteogenesis. Previous research has found that alginate hydrogels that sustain the release of Mg^2+^ contributed to the infiltration and activation of CD68^+^ macrophages in bone defects during the early stages of bone healing [22, 23]. They also found that Mg^2+^ promoted the expression of the transient receptor potential cation channel M7 (TRPM7) and nuclear translocation of TRPM7 cleaved kinase fragments (M7CKs), leading to the differentiation of macrophages into the osteogenic subtype as well as promoting bone marrow recruitment and osteogenic differentiation.

Del Giorno et al [24] found that magnesium screws can induce high expression of vascular endothelial growth factor around the implant in the rat bone marrow cavity. Yu-Xiong Su et al [25] found that magnesium implants promoted angiogenesis and osteogenesis in rats with medication-related osteonecrosis of the jaw (MRONJ). Therefore, we believe that the effect of magnesium screws in improving vascularized bone grafts is attributed to the combination of promoting osteogenesis and angiogenesis.

In the present study, we found that the improvement of Harris score was more significant in Group A than Group B and C. This indicates that the application of biodegradable magnesium screw to fix the iliac bone graft could promote the healing of the bone and facilitate recovery.

#### Biodegradable magnesium fixation is more reliable

Han et al [26] used high purity magnesium screws for internal fixation of femoral condylar fractures and found that although the high purity magnesium screws dissolved slightly faster at the fracture gap, they did not affect the fixation strength of the screws and the fractures healed without significant displacement. In this study (post-operative and follow-up), the iliac flap fixed with the biodegradable magnesium screw group did not move or dislodge, and it showed excellent fixation strength.

Mild displacement of the bone graft occurred in two patients in Group C during follow-up. In the biodegradable magnesium screw group, there was no displacement of the grafted bone. These results suggest that the use of biodegradable magnesium screws for fixation of the iliac graft is an effective method to avoid displacement of the implanted bone flap.

#### Biodegradable magnesium screws are self-degrading in vivo

Magnesium ions are absorbed to form new bone in human body [27]. Compared to other tissues, the tissue fluid circulation in the bone tissue is slow and therefore requires a relatively long degradation time for magnesium screws. If biodegradable magnesium screw degrades too quickly, they are likely to cause a local magnesium ion excess, hydrogen build-up and a high alkaline environment. The current approach to reducing the degradation rate of magnesium-based material is to increase the purity of the magnesium screw. So we used high purified magnesium in this study. Patients had normal serum calcium, magnesium and phosphorus in Group A. Besides, we found that high-purity biodegradable magnesium screws did not release other harmful metal elements during degradation.

### Advantages of DCE-MRI in assessing blood supply and the role of magnesium screws in promoting angiogenesis

The pathogenesis of non-traumatic osteonecrosis remains unknown; however, alterations in intramedullary circulation are known to play a role. Narrowing or occlusion of blood vessels within the femoral head is a direct cause of non-traumatic femoral osteonecrosis. For example, in patients with hormone-induced osteonecrosis, glucocorticoids can cause adipocyte hypertrophy, which leads to the compression of venous sinuses and reduced blood flow to the femoral head [28].

Dynamic Enhanced MRI (DCE-MRI) is a minimally invasive technique for assessing the perfusion status of a lesion. This technique reflects the distribution of microvasculature and capillary perfusion and is used to assess local tissue viability and function, allowing for a more specific assessment of the perfusion status of tissues and organs. Since conventional X-ray, CT and MRI cannot assess blood supply, and DSA (digital silhouette angiography) is more invasive (patients need to be hospitalised for follow-up examinations), the use of DCE-MRI (which allows follow-up examinations to be done on an outpatient basis without the need for hospitalisation) to assess the blood supply to the femoral head has significant advantages [29, 30]

In this study, we delineated regions of interest (ROI) in the necrotic, repaired, and greater trochanteric regions and created time-intensity (TIC) curves. We analysed the results of the DCE-MRI images and time-intensity (TIC) curves for the three groups of patients in the following three ways.

1. The preoperative DCE-MRI images and TIC curves showed that the microcirculatory perfusion in the repair-responsive zone was significantly higher than that in the greater trochanter in the three groups of patients, reflecting enhanced blood flow velocity, capillary density and permeability in the repair-responsive zone compared to the normal bone marrow, and this enhancement may be related to the massive formation of neovascularization in the repair area. In contrast, microcirculatory perfusion in the necrotic area was lower than that in the greater trochanter, reflecting a decrease in blood flow velocity, capillary density and permeability in the necrotic area compared to the normal bone marrow, and a disruption of normal microcirculation in the bone.
2. The postoperative DCE-MRI images and TIC curves showed that the microcirculatory perfusion in the repair-responsive area was lower in the three groups compared to the preoperative period, while the microcirculatory perfusion in the necrotic area was higher and closer to the normal microcirculatory perfusion in the greater trochanter. This suggests that the iliac flap transfer with vascular tip improves the blood supply to the necrotic area of the femoral head and regulates the abnormal hyperperfusion in the repaired area.
3. Comparison of preoperative and postoperative DCE-MRI images and TIC curves between the three groups revealed that more neovascularization was observed around the implantation area in the existing cases in the group using the biodegradable magnesium nail after surgery and that the TIC pattern in the necrotic and repaired areas was more normalized in this group of patients than in the control groups (Group B and C). Thus, (we hypothesize) the use of biodegradable magnesium nails for fixation of bone flaps improves microcirculation and enhances vascularization of the reconstructed femoral head, and also provides new clinical data to support the pro-angiogenic effect of biodegradable magnesium nails.

### Limitations and next plan of this study

Several limitations were identified in this study. Firstly, the sample size of 36 patients was relatively small and may not have provided sufficient statistical power. Secondly, although local pneumatization has been reported in the literature as a potential risk of magnesium screw implantation, we did not observe this phenomenon in our study, likely due to the short 6-month follow-up period. Thirdly, the presence of unilateral and bilateral hip lesions in Group A, B and C was not considered as a variable in the controlled trial, and its potential impact on study outcomes was not assessed. Fourth, the current method of semi-quantitative analysis of blood supply to the femoral head utilizing DCE-MRI may be susceptible to factors such as hemodynamics and vascular permeability, and may be influenced by parameter settings.

In future studies, we plan to conduct a longer follow-up and include a larger, more diverse patient population. We will also perform more quantitative analyses to more precisely evaluate the effect of magnesium screws on promoting angiogenesis.

## Conclusions

Our study determined that PVIBGT effectively improved blood supply to the femoral head. We propose that biodegradable magnesium screws offer two primary advantages compared to other methods. Firstly, when compared to the direct embedding method, biodegradable magnesium screws provide better fixation and reduce the risk of fixation loosening, which could potentially lead to hip preservation failure. Secondly, compared to traditional titanium screws, magnesium screws can safely degrade in vivo while also promoting angiogenesis.

## Data Availability

The datasets used during the current study are available from the corresponding author on reasonable request.

## Abbreviations

ONFH: Osteonecrosis of the femoral head
PVIGBT: Pedicled vascularized iliac bone graft transfer
DCE-MRI: Dynamic contrast-enhanced magnetic resonance imaging
TRPM7: Transient receptor potential cation channel M7
